# Supplementary material for: SyNDI: synchronous network data integration framework
Source: BMC Bioinformatics. 2018 Nov 6;19:403. doi: 10.1186/s12859-018-2426-5 (PMC6219086; doi:10.1186/s12859-018-2426-5)
Supplement: Supplementary file 5 — Files of the example presented in Additional file 4. This additional file is a zip package that contains all files used in Additional file 4. Detailed descriptions of each of these files are presented in a README file included in this zip package. (GZ 30411 kb) [file 12859_2018_2426_MOESM5_ESM.gz › SyNDITools/Meme2Fimo/meme_4.9.0_4.tar.gz!/meme_4.9.0/website/html/mcast-intro.html]

MCAST - Introduction


|  |  |
| --- | --- |
|  | MEME Suite The MEME Suite web application requires the use of JavaScript   Javascript doesn't seem to be available on your browser.  Introduction  `MCAST` searches a sequence database for statistically significant clusters of non-overlapping "hits" to the motifs in a query.  A "hit" is a sequence position that is sufficiently similar to a motif in the query. To be a hit, the *p*-value of the motif alignment score must be less than the significance threshold, *pthresh* (see optiontal input *p*-value threshold, below). The alignment of the motif and the sequence position is done **without** gaps. To compute the *p-*value of a motif alignment score, `MCAST` assumes that the sequences in the database were generated by a 0-order Markov process. `MCAST` searches for hits on both the sequences given in the database, and their reverse complements.  A cluster of non-overlapping hits is called a "match". The user can specify the maximum allowed distance between the hits in a match (see optional input maximum motif gap below). Two hits separated by more than the maximum allowed gap will be reported in separate matches.  `MCAST` searches for all of the matches between the query and the sequences in the database. Each match is assigned an *E*-value, and matches that score below an *E*-value threshold are printed in order of increasing *E*-value (see optional input *E*-value threshold below).  The *p-*value of a hit is converted to a "p-score" in order to compute the total score of the match it participates in. The p-score for a hit with *p-*value *p* is  *S = -log_2_(p/pthresh),*  where the significance threshold *pthresh* may be specified by the user (see optional input motif *p*-threshold below. The total score of a match is the sum of the p-scores of the hits making up the match. `MCAST` finds the matches with the maximum match scores.  In order for *E*-values to be computed by `MCAST`, at least 100 matches must be found. If there are too few sequences in the database, or if certain other options are made to stringent (see Options, below), too few matches may exist for *E*-values to be computed. In this case, the results are sorted by match score, the *E*-value column is set to "NaN" and all matches are printed.  A full description of the algorithm is found in:  Bailey and Noble. "Searching for statistically significant regulatory modules." *Bioinformatics (Proceedings of the European Conference on Computational Biology)*. 19(Suppl. 2):ii16-ii25, 2003.  Required MCAST Inputs  Three inputs must be provided on the MCAST web page:  1. An e-mail address where the notification of job completion can be sent.    You specify the e-mail address in the two text boxes labeled "e-mail address".    The e-mail address must be entered twice to reduce the amount of    undeliverable mail caused by typographic errors. 2. A MEME output file,    containing the descriptions of one or more motifs.    You can select a file to be uploaded from your computer by clicking on the    "Browse ..." button under the "Your motif file" label. 3. A sequence database to be searched. You can choose a sequence file to be    uploaded from your computer bu clicking on the "Browse ..." button under the "Your    FASTA sequence file" label.    Alternatively, you can select one of the supported databases maintained on    the MEME Suite web site:    first select the category of the    sequence database from the "Category" drop-down list, then choose one of    the supported databases listed in the "Database" drop-down list.   Optional MCAST inputs  The MCAST web page accepts four optional inputs:  1. A threshold p-value. Motif occurrences with p-values below the threshold    will not be considered in scoring matches (defaults to `5e-4`). 2. A motif gap maximum. The maximum allowed distance between adjacent motif    hits in a match (defaults to 50). 3. The E-value threshold. Matches whose E-value is less then the threshold    will not be reported (defaults to 10). 4. The pseudocount weight. A pseudocount is added to each count in the    motif matrix. The pseudocount is determined by multiplying the background    frequency by this weight (defaults to 4).   The contents of the required and optional fields can be cleared by clicking on the "Clear Input" button. Submitting an MCAST job  After filling in the required and optional inputs on the MCAST web page, the job can be submitted for processing by clicking on the "Start Search" button. MCAST output  The result of submitting your job will be a summary page that briefly describes your job's input, or reports any critical errors. If there are no errors, then the page will also contain a link to your job's results. The results page will report the full command that was run and will include links to the MCAST output, or to any error messages that were generated. The MCAST output will be an HTML file (sample) containing a table of clusters of the input motifs, sorted by E-value, followed by alignment diagrams showing the motif hits in each cluster. |
